# Effects of Coconut Water on Retina in Diabetic Rats

**DOI:** 10.1155/2020/9450634

**Published:** 2020-01-28

**Authors:** Xiaohua Zhang, Li Peng, Yanan Dai, Xia Sheng, Shaomei Chen, Qing Xie

**Affiliations:** ^1^Department of Ophthalmology, Central South University Xiangya School of Medicine Affiliated Haikou Hospital, Haikou, Hainan, China; ^2^Department of Ophthalmology, The Second Xiangya Hospital, Central South University, Changsha, Hunan, China; ^3^Department of Ophthalmology, Changsha Central Hospital, Changsha, Hunan, China

## Abstract

Coconut water (CW) is a natural aseptic nutritious beverage, containing several biologically active compounds. This study aimed to determine the antiretinopathy effects of CW on diabetic Sprague Dawley (SD) rats using streptozotocin (STZ) and explore its potential mechanism. After allowing the rats to acclimatize for 7 days, 48 healthy adult male SD rats were selected and randomly divided into 4 groups, involving control (Ctrl), diabetic rats (DM), diabetic rats treated with CW (DM-CW), and diabetic rats treated with glibenclamide (DM-Gli). The diabetic models were established by an intraperitoneal injection of STZ (60 mg/kg). The Ctrl group was injected with an equal volume of sodium citrate solution. The experiment was totally conducted during 20 weeks, and then, all rats were sacrificed. The serum levels of superoxide dismutase (SOD), malondialdehyde (MDA), and glutathione peroxidase (GSH-Px) were measured; additionally, the activities of interleukin-6 (IL-6) and intercellular adhesion molecule-1 (ICAM-1) in the retina were investigated using biochemical assays. Hematoxylin and eosin (H&E) staining was performed to observe pathological changes of retinal tissues. In presence of treatment with CW, serum level of MDA was decreased, while serum levels of SOD and GSH-Px were increased; besides, the activities of IL-6 and ICAM-1 in the retina were reduced compared with the DM group. The antiretinopathy feature of CW was confirmed by the increased number of neurons in the ganglion cell layer (GCL), total retina thickness (TRT), and thickness of the retinal nuclear layer (RNL) in diabetic rats. CW can be protective against diabetic retinopathy (DR), and its effects are comparable to Gli. The possible underlying mechanism may be partly explained by decreasing oxidative stress and anti-inflammatory activities in the retina. However, further research should be conducted to reveal the exact mechanism.

## 1. Introduction

Chronic hyperglycemia of diabetes is associated with long-term damage, dysfunction, and failure of different organs, especially eyes [1]. The World Health Organization (WHO) has estimated that the prevalence of diabetes among adults (age, 20–79 years) will be globally 7.7% in 2030, involving 439 million adults [2]. Besides, in the United States, the prevalence will reach 33% by 2050 [3]. Diabetic retinopathy (DR) is a chronic, progressive sight-threatening disease of the retinal microvasculature, presenting a dynamic process from nonproliferative retinopathy to proliferative retinopathy, and it has become the leading cause of blindness in young adults in developed countries [4]. With the extension of the course of disease, the incidence of DR increases, and the rate and speed of its development from nonproliferative retinopathy to proliferative retinopathy increase as well. In addition to the early control of blood glucose, therapies include laser treatment, vitreous cut surgery, and application of antivascular endothelial growth factor (VEGF); although these therapeutic approaches have been found to be partly successful, however, they still have remained unsatisfactory because they do not prevent advances in retinopathy from early stage to late stages, as well as causing serious financial burden to patients [5]. Therefore, early intervention is of great significance to prevent or delay the development of DR, as well as being an important measure to prevent and treat blindness.

Multiple mechanisms are involved in the process of DR formation. A study showed that the cellular components of the retina are highly compacted, while they are very susceptible to the hyperglycemic environment. The retina responds to the hyperglycemic environment through a number of biochemical changes, e.g., increased oxidative stress, causing disturbance of the internal environment. Oxidative stress is taken as one of the crucial contributors in the pathogenesis of DR into consideration [6]. In addition to oxidative stress, several scholars found that inflammatory response plays a pivotal role in DR [7]. A clinical study reported that, when cells were soaked in a high-glucose environment for a period of time, the control of blood glucose could not fully inhibit the inflammatory response caused by previous high-glucose exposure, which was called “metabolic memory.” [8] A number of scholars also confirmed that the pathological features and development process of DR are associated with low-grade chronic inflammation, and interleukin-6 (IL-6) and intercellular adhesive molecule-1 (ICAM-1) are two important inflammatory cytokines induced by high-glucose levels, leading to diabetes-related complications [9, 10].

Coconut water (CW) is a clear, nutritive liquid obtained from the endosperm of coconuts, and its electrolyte composition and proportion are similar to human plasma and have strong antioxidant activity [11, 12]. CW contains a variety of biological active ingredients; numerous studies showed that CW has a protective potential against diabetes, which can reduce diabetes-related complications. It possesses cardioprotective, hepatoprotective, hypolipidemic, and antihypertensive properties in experimental animals and hypoglycemic, antithrombotic, and antioxidant activities in induced diabetic rats [13–18].

This study aimed to assess the protective effects of CW on the retina of diabetic rats induced by streptozotocin (STZ) and also explore the possible pathogenesis.

## 2. Materials and Methods

### 2.1. Experimental Animals and Preparation of CW

Healthy adult male Sprague Dawley (SD) rats without fundus disease, weighing between 300–400 g, were used for analysis. They were purchased from Tianqin Biotechnology Co., Ltd. (Changsha, China). The rats were maintained in an air-conditioned animal house under a normal day/night cycle and fed with a normal rodent chow diet and water *ad libitum*. All animal procedures and experiments were conducted in accordance with the Chinese guidelines, and the study protocol was approved by the Institutional Review Board of Haikou People's Hospital (Haikou, China).

CW was derived from mature coconuts with the age of 6–8 months (*Cocos nucifera* L., Arecaceae), which was harvested from the coconut trees grown on Wenchang (Hainan, China). The coconuts were dehusked, and liquid endosperm was collected. The collected CW was stored in a refrigerator at 4°C for the experiment.

### 2.2. Experiments

#### 2.2.1. Grouping and Establishment of Rat Diabetic Models

After allowing 48 rats to acclimatize for 7 days, all rats were overnight-fasted, and according to a random number table, those rats were randomly divided into four groups, including control (Ctrl), diabetic rats (DM), diabetic rats treated with CW (DM-CW), and diabetic rats treated with glibenclamide (DM-Gli) by a single intraperitoneal injection of 1% (W/V) STZ (60 mg/kg) in 0.02 M citrate buffer (pH 4.5) except for the Ctrl group which injected with the vehicle only (*n* = 12 rats for each group) [19]. Moreover, 72 h after STZ injection, fasting blood glucose (FBG) levels were measured by taking blood from the tail, and animals with blood glucose levels of >16.7 mmol/L were taken diabetic into consideration. A total of 33 rats were successfully modeled (*n* = 11 rats for each group). Simultaneously, in order to maintain consistent quantity, 1 rat was randomly removed from the Ctrl group. As mentioned earlier, all groups were continually acclimatized for 1 month through feeding with normal rodent chow diet and water *ad libitum*. Besides, after 1 month, all groups were treated as follows (the whole observation period was 20 weeks):

  Group I: Ctrl, *n* = 11: normal rodent chow diet and water *ad libitum*
  Group II: DM, *n* = 11: normal rodent chow diet and water *ad libitum*
  Group III: DM + CW, *n* = 11: normal rodent chow diet and CW *ad libitum*
  Group IV: DM + Gli, *n* = 11: normal rodent chow diet and water *ad libitum* + daily orally given Gli at a dose of 0.6 mg/kg [17]

#### 2.2.2. Harvesting the Samples

At the end of the experiment, animals were fasted overnight, and they were fully anaesthetized with 10% chlorine hydrate. The bilateral eyeballs were removed after anesthesia. The left eyeballs were cut along the angle of the sclera edge on ice using a microscope, and then, retinal tissues were collected, washed with 0.9% saline, dried with a filter paper, weighted before putting into the cryopreservation tube, and finally stored at −80°C for the next experiment. The whole eyeballs on the right were fixed in 10% buffered formalin overnight and after that embedded into paraffin for later use. After removing the eyeballs, the belly line was incised, separated bluntly to reveal the abdominal aorta, and the arterial blood was collected and centrifuged to obtain the supernatant, in which the supernatant was stored at −80°C.

#### 2.2.3. Observation and Detection of Experimental Indicators

*Histopathological Analysis of the Rat Retina.* The collected eyeballs were embedded into paraffin, sectioned to 3 *μ*m thickness, and stained with hematoxylin and eosin (H&E). Light microscopy was used to investigate histomorphology of the retina (BX51; Olympus Corp., Tokyo, Japan).

Microscopic evaluation of the retina included measurement of total retina thickness (TRT), thickness of the retinal nuclear layer (RNL), and the number of neurons in the ganglion cell layer (GCL). The number of neurons in the GCL was quantified by counting cells from the temporal to the nasal, and the counted length was 280 *μ*m in the present study. Retinal thickness was measured at 4 points at the posterior of the retina, two on both sides of the optic nerve and were approximately 200 to 300 *μ*m. Three slices were taken from paraffin, and each experiment was repeated three times. The measurements were then averaged to yield a measurement for each rat [20].

*Biochemical Analysis.* A certain amount of phosphate buffer solution (0.1 M, pH 7.4) was added to the retinal tissues to make a solution with a mass fraction of 50%. Each tissue was homogenized after shearing and centrifuged at 3000 rpm for 20 min at 4°C. Then, the supernatant was separated and used for the determination of biochemical parameters.

The supernatant obtained from the blood was processed for detecting the levels of glutathione peroxidase (GSH-Px), superoxide dismutase (SOD), and malondialdehyde (MDA), and the supernatant from the retina was used to measure the levels of ICAM-1 and IL-6 by using commercially available enzyme-linked immunosorbent assay (ELISA) kits (Shanghai Jianglai Biotechnology Co., Ltd., Shanghai, China). Our expected levels were determined by the double-antibody sandwich ELISA (DAS-ELISA) method. The value of optical density (OD) was measured at 450 nm wavelength with an enzyme marker, and our expected concentration was calculated by a standard curve.

### 2.3. Statistical Analysis

Statistical analysis was undertaken via SPSS 22.0 software (IBM, Armonk, NY, USA). Data were expressed as mean ± standard deviation (SD). One-way analysis of variance (ANOVA) and Tukey's Honest Significant Difference test were used to compare differences among the groups. *P* < 0.05 was considered statistically significant.

## 3. Results

### 3.1. Effects of CW on Retinopathy in Diabetic Rats

**Figure 1:**
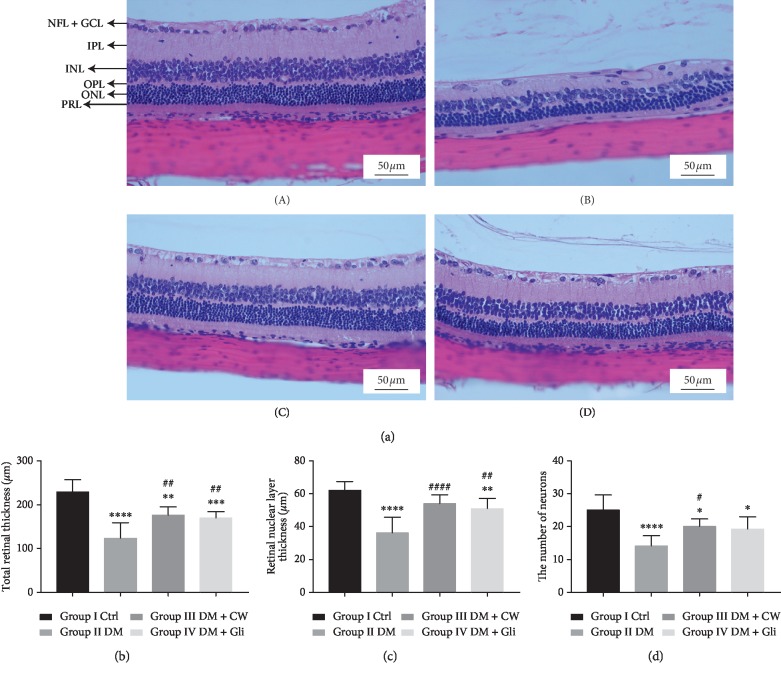
(a) Light microscopy of the transverse section of the eyeball (3 *μ*m thickness), stained with H&E, with magnification of 400x, in order to determine pathological changes in the retina at the end of experiment (week 20). A control rats; B diabetic rats which received normal water; C diabetic rats which received CW; D diabetic rats which received Gli. Abbreviations: NFL, nerve fiber layer; GCL, ganglion cell layer; IPL, inner plexiform layer; INL, inner nuclear layer; OPL, outer plexiform layer; ONL, outer nuclear layer; PRL, photoreceptor layer. (b) Total retina thickness (TRT) in control and diabetic rats (DM), which received either normal water or CW or Gli for 20 weeks. ^*∗∗*^,^*∗∗∗*^,^*∗∗∗∗*^*P* < 0.01, 0.001,  and 0.0001, respectively, compared with the control group; ^##^*P* < 0.01 compared with the diabetic group. (c) The thickness of the retinal nuclear layer (RNL) for control and diabetic rats (DM), which received either normal water or CW or Gli for 20 weeks. ^*∗∗*^,^*∗∗∗∗*^*P* < 0.01 and 0.0001, respectively, compared with the control group; ^##^,^####^*P* < 0.01 and 0.0001, respectively, compared with the diabetic group. (d) The number of cells in the ganglion cell layer (GCL) in control and diabetic rats (DM), which received either normal water or CW or Gli for 20 weeks. ^*∗*^,^*∗∗∗∗*^*P* < 0.05 and 0.0001, respectively, compared with the control group; ^#^*P* < 0.005 compared with the diabetic group.

The effects of CW on histopathological changes of the retina are shown in Figure 1(a) (A). Retinas of rats in the Ctrl group had a GCL, in which the cells were densely packed, except for blood vessels, and there was evidence of an intervening space between cells. The cells in the nuclear layer were not only abundant but also densely packed (Figure 1(a) (A)). At the end of the experiment, the number of neurons in GCL, TRT, and RNL thickness was significantly decreased in the DM group, which received normal water (*P* < 0.0001 compared with the Ctrl group). However, as illustrated in Figures 1(b)–1(d), the number of neurons in GCL, TRT, and RNL was attenuated in the DM group, which received CW (*P*  value < 0.05, 0.01,  and  0.0001, respectively, compared with the DM group). As displayed in Figure 1(d), it can be concluded that CW outperformed in preventing reduction of the number of neurons in GCL compared with Gli.

### 3.2. Effects of CW on Oxidative Stress Markers

**Figure 2:**
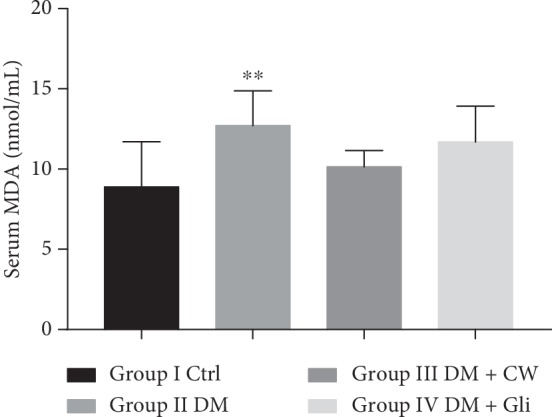
The serum level of malondialdehyde (MDA) (nmol/ml) in control and diabetic rats (DM), which received either normal water or CW or Gli for 20 weeks. ^*∗∗*^*P* < 0.01 compared with the control group.

**Figure 3:**
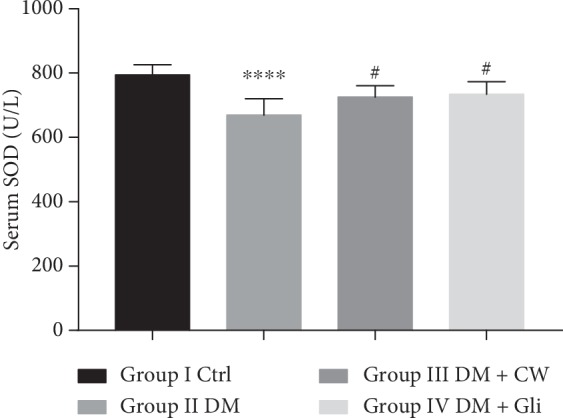
The serum level of superoxide dismutase (SOD) (U/L) in control and diabetic rats (DM), which received either normal water or CW or Gli for 20 weeks. ^*∗∗∗∗*^*P* < 0.0001 compared with the control group, and ^#^*P* < 0.05 compared with the diabetic group.

**Figure 4:**
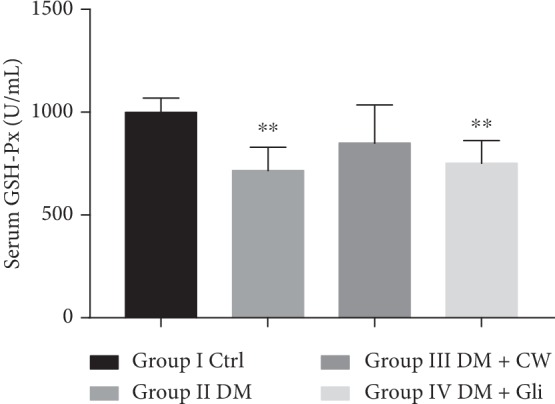
The serum level of glutathione peroxidase (GSH-Px) (U/mL) in control and diabetic rats (DM), which received either normal water or CW or Gli for 20 weeks. ^*∗∗*^*P* < 0.01 compared with the control group.

With respect to the substantial role of oxidative stress on retinopathy, the level of MDA and the activities of SOD and GSH-Px in serum were analyzed to assess the effects of CW on oxidative stress markers. As depicted in Figures 2–4, the level of MDA was significantly increased in the DM group, while the activities of SOD and GSH-Px were decreased (*P* < 0.01, 0.0001,  and 0.01, respectively, compared with the Ctrl group). Treatment with CW remarkably mitigated the decline of SOD activities, which induced by diabetes mellitus (*P* < 0.05 compared with rats in the DM group), and there was no significant increase or decline in the level of MDA or activities of GSH-Px after treatment with CW compared with the Ctrl group (*P* > 0.05 for both). As illustrated in Figure 4, CW outperformed in reducing the decline of activities of GSH-Px compared with Gli.

### 3.3. Effects of CW on Retinal Inflammatory Factors

**Figure 5:**
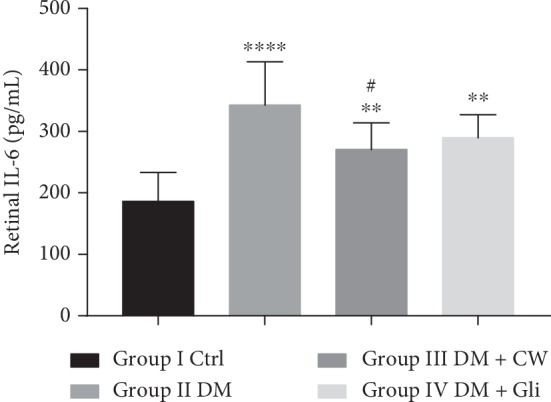
The level of interleukin-6 (IL-6) (pg/mL) in the retina of control and diabetic rats (DM), which received either normal water or CW or Gli for 20 weeks. ^*∗∗*^,^*∗∗∗∗*^*P* < 0.01 and 0.0001 compared with the control group, and ^#^*P* < 0.05 compared with the diabetic group.

**Figure 6:**
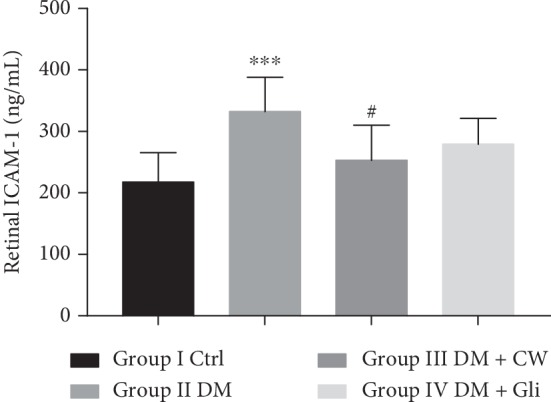
The level of intercellular adhesion molecule-1 (ICAM-1) (ng/mL) in the retina of control and diabetic rats (DM), which received either normal water or CW or Gli for 20 weeks. ^*∗∗∗*^*P* < 0.001 compared with the control group, and ^#^*P* < 0.05 compared with the diabetic group.

In addition to oxidative stress, the inflammatory cytokines also play a crucial role in the development of DR. Therefore, whether CW has protective effects on the inflammatory response of the retina was also investigated by studying the levels of IL-6 and ICAM-1 in the retina, and the results are shown in Figures 5 and 6. It was revealed that the levels of IL-6 and ICAM-1 in the retina of rats in the DM group were increased (*P* < 0.0001 and 0.001 compared with the Ctrl group). Besides, CW could significantly decrease the elevation of IL-6 and ICAM-1 levels induced by diabetes mellitus in the rat retina (*P* < 0.05 and 0.05 compared with the DM group). In contrast, Gli did not significantly attenuate the increase of IL-6 level (*P* < 0.01 compared with the Ctrl group, and *P* > 0.05 compared with the DM group).

## 4. Discussion

DR is an important cause of blindness; however, the pathogenesis of DR formation is highly complicated and needs to be further studied. Thus, conducting research on prevention and treatment of DR is of great significance. In the current study, we first investigated the effects of CW on the DR progression in STZ-induced diabetic rats, and it was found that CW could effectively protect the development of DR.

To our knowledge, among several causes, oxidative stress plays a substantial role in retinopathy. Cells have an antioxidant defense system against harmful effects of oxidant compounds, and MDA, SOD, and GSH-Px are classical indicators to evaluate oxidative stress level [21]. We detected the effects of CW and Gli on the serum MDA level, as well as SOD and GSH-Px activities. The results were consistent with findings reported by previous research studies in diabetic patients, in which the DM group expressed decreased SOD and GSH-Px activities and increased MDA level in contrast to the Ctrl group [22]. After treatment with CW, MDA level was decreased, while the SOD and GSH-Px activities were enhanced, and CW outperformed in reducing the decline of GSH-Px activities compared with Gli. These results suggested that treatment with CW might protect against oxidative stress causing damage to the retina. Being consistent with previous reports, CW influences free-radical scavenging properties [23].

In addition to the oxidative stress aggravating DR as mentioned above, there is increasing evidence that inflammation plays a significant role in the pathogenesis of retinopathy [7]. Besides, tumor necrosis factor (TNF), IL-1, and IL-6 are common proinflammatory cytokines, and they are found in serum, vitreous, or retinas of diabetic patients or rats [24, 25]. Hence, a number of scholars suggested that diabetes is a chronic, low-grade inflammatory, and autoimmune disease [25]. High level of blood glucose can induce leukocyte adhesion to vascular endothelial cells, and this may cause destruction of the blood retinal barrier (BRB) and aggravate microcirculation, resulting in the hypoxia state of the retina that stimulates the expression level of VEGF. Increased VEGF upregulates the expression levels of IL-1*β* and adhesion factors (e.g., ICAM-1 and VCAM-1), creating a vicious cycle, eventually contributing to the development of DR [26, 27]. As a main proinflammatory mediator, IL-6 has a direct toxic effect on islet cells and can upregulate the expression level of ICAM-1, mediated by IL-1-induced by reactive oxygen species, leading to further damage to the epithelium, as well as accelerating the onset of diabetes [28]. The structural failure of BRB might also lead to neuronal cell loss, especially in the GCL, playing a crucial role in the reduction of outer nuclear layer (ONL) and TRT [29, 30]. The degeneration of retinal ganglion cells is affected by numerous factors, including polyol pathway, oxidative stress, inflammation, or exposure to advanced glycation end products [31, 32]. The results of H&E staining showed that treatment with CW can attenuate the reduction of TRT, thickness of RNL, and loss of neurons in GCL in rats in the DM group, and the structure of each layer of the retina was relatively complete. Similar to other reported results, the present study also indicated that the levels of ICAM-1 and IL-6 in rats in DM group were significantly higher than those of the Ctrl group, suggesting that 5-month-old diabetic rats developed inflammatory damage to the endothelium. However, after intervention with CW, the levels of ICAM-1 and IL-6 were notably reduced. These results revealed that CW can inhibit the early adhesion of white blood cells, reduce inflammatory damage to the retina, improve microcirculation, and alleviate and delay the progression of DR.

It is noteworthy that CW is rich in ascorbic acid, cysteine, phenolic compounds, and L-arginine, which are closely associated with biological activities and pharmacological effects of CW. The abovementioned biological components are excellent free-radical scavengers, protecting the organs through oxidation resistance [14, 16, 33, 34]. Numerous studies concentrated on the role of L-arginine in diabetic rats, as well as being an important modulator of glucose metabolism and promoting an increase in polyamase in the pancreas of diabetic rats to enhance recovery of the endocrine pancreatic function, in which L-arginine may play a role in the restoration of glutathione peroxidase activity in diabetic rats [35, 36]. L-arginine has been reported due to its antithrombotic feature and increased fibrinolytic activity, which can improve endothelium-dependent vasodilatation ability by suppression of platelet aggregation and increased levels of NO [13]. Anti-inflammatory properties of CW have also been reported, in which the observed antipyretic and anti-inflammatory activities of CW may be due to the inhibition of prostaglandin release or blocking the enzyme, cyclo-oxygenase, reflecting that the exact mechanism remains elusive [37].

## 5. Conclusions

It can be concluded that CW can be protective against DR. The possible underlying mechanism may be partly explained by decreasing oxidative stress and anti-inflammatory activities in the retina. Although a variety of chemical tests or animal experiments have demonstrated that several chemical active compounds can be used to explain the beneficial effects of CW, e.g., ascorbic acid, cysteine, phenolic compounds, and L-arginine; however, further research needs to be conducted to indicate the exact mechanism of antiretinopathy effects of CW.

## Data Availability

The data used to support the findings of this study are available from the corresponding author upon request.
